# Opportunistic infections among schoolchildren who were on antiretroviral therapy in Ethiopia: a systematic review and meta-analysis

**DOI:** 10.3389/fped.2024.1255111

**Published:** 2024-11-22

**Authors:** Molla Yigzaw Birhanu, Animut Takele Telayneh, Abere Kassie, Eniyew Tegegne, Selamawit Shita Jemberie

**Affiliations:** ^1^ Department of Public Health, College of Health Sciences, Debre Markos University, Debre Markos, Ethiopia; ^2^ Department of Pediatric and Child Health, College of Health Sciences, Debre Markos University, Debre Markos, Ethiopia; ^3^ Department of Environmental Science, College of Health Sciences, Debre Markos University, Debre Markos, Ethiopia; ^4^ Department of Midwifery, College of Health Sciences, Debre Markos University, Debre Markos, Ethiopia

**Keywords:** schoolchildren, opportunistic infections, onset and predictors, children on ART, Ethiopia

## Abstract

**Introduction:**

The most common and severe cause of morbidity and mortality among HIV- positive children is opportunistic infections (OIs). All HIV-infected children are at risk of developing a variety of OIs. Healthcare workers, programmers, and other stakeholders are in doubt about using the onset and predictors of OIs among schoolchildren on antiretroviral therapy (ART) due to the presence of conflicting results found in the primary studies. Hence, this study was conducted to provide a single figure of onset and specific predictors of OIs by overcoming the existing heterogeneity in Ethiopia.

**Methods:**

The included studies were searched from different national and international databases systematically. The included studies were cohort in design and published in English between 2015 and 2022. The data were extracted using a validated Microsoft Excel tool after the quality of the included studies was assured. The extracted data were exported to Stata Version 17.0 for further management and analysis. The presence of heterogeneity across studies was checked using the Chi-square test and quantified using the *I*^2^ test. Various methods, including forest plots, publication bias assessment, sensitivity tests, subgroup analysis, and meta-regression, were employed to determine the source of heterogeneity, but none were successful. The overall onset of OIs was estimated by pooling the incidence of primary studies using a random-effects meta-analysis model. The predictors were identified using meta-regression and the presence of significant association was declared using a *p*-value of 0.05 with 95% CI. The strength of association was reported using an adjusted hazard ratio with 95% CI.

**Results:**

Eleven studies were included in this systematic review and meta-analysis. The onset of OIs among schoolchildren on ART in Ethiopia was 5.58 (95% CI: 4.50, 6.67) per 100 children-years of OI-free observations. Those children who had no parents had a 1.41 (95% CI: 1.10, 1.80) times higher chance of getting OIs when compared with those children having one or both parents. Children who had poor ART adherence had a 2.96 (95% CI: 1.66, 5.29) times higher chance of experiencing OIs than children who had good ART adherence. Finally, the chance of experiencing OIs among rural children was 2.15 (95% CI: 1.63, 2.83) times higher than their counterparts in Ethiopia.

**Conclusions:**

Three in every 33 schoolchildren on ART developed OIs in Ethiopia. Predictors of OIs included schoolchildren without parents, those with poor adherence to ART, and rural residents. This suggests that social support, medication adherence, and access to healthcare services may play important roles in preventing and controlling OIs among schoolchildren living with HIV in rural areas.

## Introduction

Acquired immunodeficiency syndrome (AIDS) is a pandemic disease caused by the human immunodeficiency virus (HIV) (1). HIV deteriorates the human immune system and makes the body vulnerable to secondary and opportunistic infections (OIs) (2). The most common and severe cause of morbidity and mortality among HIV-positive children is opportunistic infection (OI) (3).

Opportunistic infections (OIs) are infections that are more common and severe in people with weakened immune systems, such as people living with human immunodeficiency virus (PLHIV) (4). All HIV-infected children are at risk of developing a variety of OIs in the first 6–12 months of antiretroviral therapy (ART) treatment initiation due to immune reconstitution inflammatory syndrome (IRIS) that occurs when ART is started. IRIS can occur when the immune system starts to recover on ART, leading to an inflammatory response against existing opportunistic infections, and it is most common in the first 3–6 months of ART (5).

The most common OIs in low and middle-income countries are tuberculosis (TB), oral candidiasis, varicella zoster, pneumocystis pneumonia, cryptococcus disease, tuberculosis, cytomegalovirus disease, and disseminated non-tuberculous mycobacterial infections, herpes zoster, and dermatophytes’ infections (6, 7). OIs have a significant impact on the treatment outcomes of PLHIV, causing poor quality of life, hastening disease progression, increasing medical costs, increasing the risk of treatment failure, and impairing patient response to antiretroviral therapy (ART) drugs unless they are treated as soon as possible (8, 9).

Before the development of highly effective combination antiretroviral therapy (ART) regimens, the burden of AIDS-related OIs and mortality was significantly higher, and opportunistic infections (OIs) were dramatically reduced during the era of antiretroviral therapy. However, AIDS-related morbidity and mortality remain high, particularly in developing countries (4). To reduce the occurrence of OIs among PLHIV, the World Health Organization (WHO) recommends a variety of medical interventions. These interventions include exposure reduction, chemoprophylaxis (primary/secondary), immunization, and early ART initiation (10).

Antiretroviral therapy drugs are medications that must be taken for the rest of one's life and must be monitored to ensure that maximum viral suppression is maintained. It is not a healing drug that reduces the risk of HIV-related morbidity and mortality when it has a good adherence (11). ART adherence is the extent to which individuals with HIV/AIDS consistently take their antiretroviral medication as prescribed. Adherence to ART is crucial for the successful management of HIV/AIDS, as consistent use of these medications can suppress the virus, improve immune function, and reduce the risk of transmitting the virus to others. Poor adherence to ART can lead to treatment failure, drug resistance, and progression of the disease (12).

The viral load is an important indicator of the progression of HIV infection and the effectiveness of antiretroviral therapy (ART) in controlling the virus. It is the amount of HIV present in a person's blood and is measured by counting the number of HIV RNA particles in a milliliter of blood. A high viral load indicates that the virus is actively replicating in the body, which can lead to more rapid progression of HIV disease and an increased risk of transmission to others. On the other hand, a low or undetectable viral load (less than 50 copies/ml) is a goal of HIV treatment and is effectively suppressed by ART (13).

This study was conducted due to the presence of inconsistencies in the onset of OIs across the primary studies and providing a single figure of schoolchildren on ART in Ethiopia. Therefore, the results of this study will be used to design interventions and prevention mechanisms for policymakers and healthcare workers to improve the quality of life of children living with HIV (CLHIV). Furthermore, this study will provide the importance of detecting OIs in HIV-positive children as early as possible.

**Research question**: What is the onset of opportunistic infections among schoolchildren in Ethiopia? What are the predictors of opportunistic infections among schoolchildren in Ethiopia?

**Condition:** Opportunistic infections

**Exposure**: Predictors

**Context:** Ethiopia

**Population:** Schoolchildren living with HIV on ART

## Methods

### Study area

Ethiopia is a Federal Democratic Republic with nine regional states (Afar, Amhara, Benishangul-Gumuz, Gambella, Harari, Oromia, Somali, Southern Nations Nationalities and People's Region, and Tigray) and two city administrations (Addis Ababa and Dire Dawa). It has a total area of 1,100,000 km^2^ and is divided into zones, which are further subdivided into districts, which are further subdivided into kebeles, the lowest administrative divisions (14). Ethiopia, with a population of approximately 112 million people, is Africa's second most populous country (56,010,000 females and 56,069,000 males in 2019) (15).

### Data source and search strategy

The included studies were searched from national and international databases such as (PubMed/Medline, CINAHL, Embse, Google Scholar, and ScienceDirect) with different searching strategies. Different search engines were applied, and we tried to show PubMed database searches applied as an example: (((((((((Ethiopia[Text Word]) OR (Ethiopia[MeSH Terms])) AND (Children[MeSH Terms])) OR (Child[Text Word])) OR (Adolescent[Text Word])) OR (children[MeSH Terms])) AND (Opportunistic infections[MeSH Terms])) OR (Opportunistic infections[Text Word])) AND (Incidence[Text Word])) AND (Predictors[Text Word])”. In addition to the above, the references of references (snowball) sampling tricks were implemented to have exhaustive inclusion of published articles in the study. This systematic review and meta-analysis was reported using the Preferred Reporting Items for Systematic Review and Meta-Analysis (PRISMA) guideline to keep the content validity (16) (Supplementary Table S1). Two authors (MB and SJ) worked on the search activities independently. EndNote X9 was used to retrieve and manage studies, which were accessed in a systematic search. The search strategy was performed from 5 April 2023 to 20 May 2023.

### Eligibility criteria

#### Included criteria

All cohort studies conducted on the onset and predictors of OIs among schoolchildren living with HIV on ART and published in English were included.

#### Excluded criteria

Those articles having similar titles but different outcomes were excluded.

#### Design

Systematic review and meta-analysis.

#### Screening procedure

**Figure 1:**
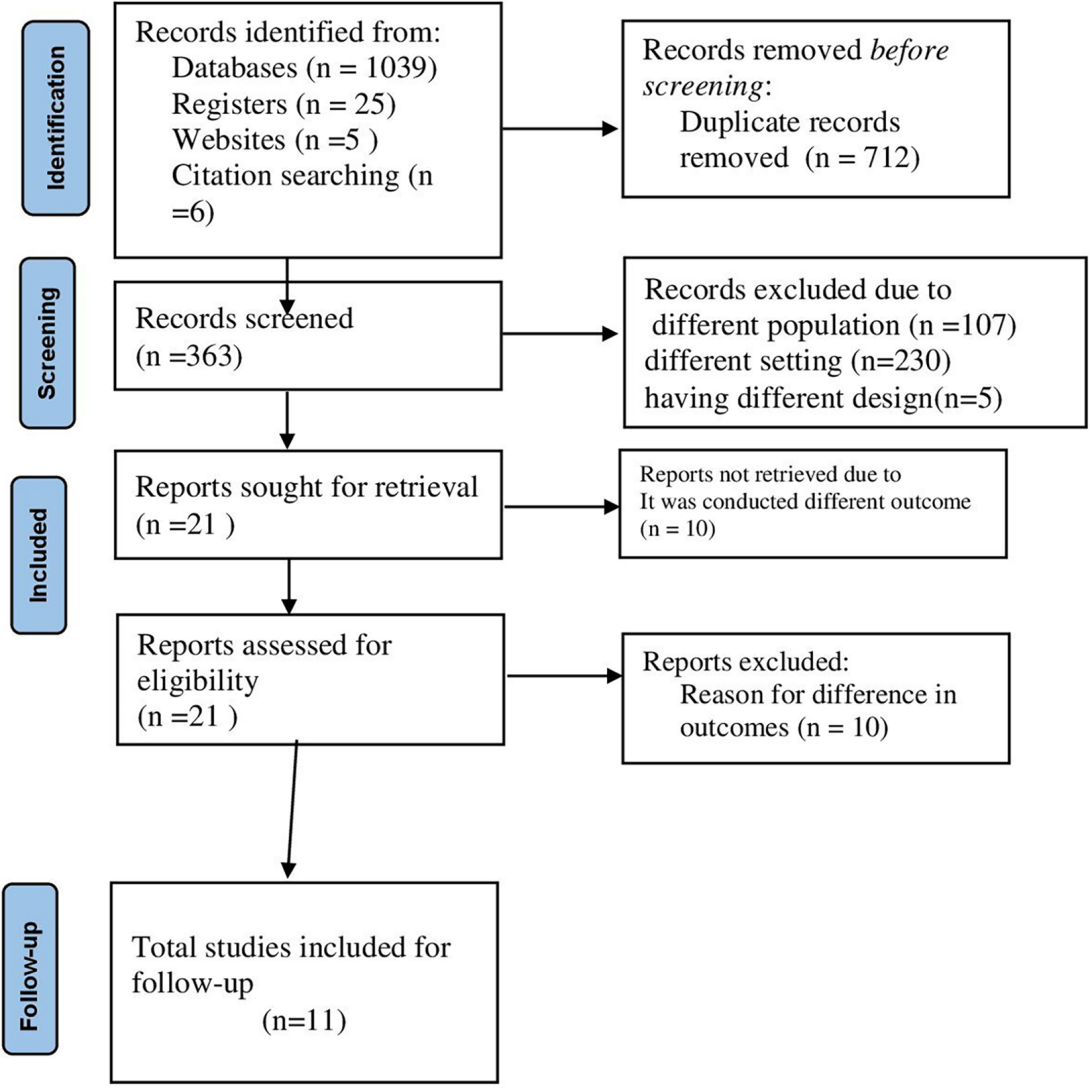
STROBE flow diagram of the included studies for OIs among Ethiopian children on ART.

After the studies were found from different sources, three authors (MB, and SJ) screened the titles and abstracts using the predefined eligibility criteria and disagreements were solved via discussion in the presence of a fourth author. The first screening was considering the titles and then followed by abstracts to check the outcome measured (Figure 1).

#### Quality assessment/risk of bias

The quality of the included studies was measured using the Newcastle–Ottawa quality assessment scale for cohort study (NOQAS). The assessment was based on selection, comparability, and outcome. As a result, studies that scored 3 or 4 stars in the selection domain AND 1 or 2 stars in the comparability domain AND 2 or 3 stars in the outcome/exposure domain were declared as “good quality”. Furthermore, articles that scored 2 stars in the selection domain AND 1 or 2 stars in the comparability domain AND 2 or 3 stars in the outcome/exposure domain were declared as “fair quality.” Finally, those articles that scored 0 or 1 star in the selection domain OR 0 stars in the comparability domain OR 0 stars in the outcome, all included articles were declared as having “good quality.”

#### Data extraction

The data were extracted using the data extraction checklist adapted from a Microsoft Excel spreadsheet. Three authors (MB, SJ, and GMB) extracted data independently and checked consistency. Disagreements between or among authors were resolved through discussion after the source of the disagreements was identified. The onset of opportunistic infections among schoolchildren, study region, year of publication, sample size, follow-up period, and first author's name were all extracted from the primary articles during the study period to compute subgroup analysis.

#### Outcome variable and measures

The onset of OIs among schoolchildren on ART was the primary outcome of interest in this systematic review and meta-analysis. It was calculated by pooling the onset of OIs from the primary studies or by dividing the total number of new cases from all studies by the total number of children-years of observations. The second outcome variable was predictors of OIs among schoolchildren on ART in Ethiopia identified using a binary meta-regression analysis model.

#### Data management and analysis

For further process and analysis, the extracted data were exported from Microsoft Excel to Stata Version 17.0 statistical software. The pooled onset of OIs was estimated using a random-effects regression model with the DerSimonian–Laird method. The standard errors were calculated from the reported estimates and population denominators using a binomial distribution assumption. The presence of heterogeneity across studies was checked using Cochran’s Q test and quantified using *I*^2^. Furthermore, the dispersion of individual results in the forest plot was used to visually assess the presence of heterogeneity. To estimate the effect size, a random-effects model based on the DerSimonian–Laird approach was used (17). A funnel plot, Egger's linear regression test, and fill and trim analysis with a *p*-value of 0.05 were used to determine the presence of publication bias (18). A 95% confidence interval was used to calculate and report an overall synthesized measure of effect size. To identify the source of heterogeneity, additional statistical analyses such as subgroup analysis and meta-regression were performed. Finally, the results are presented using texts, tables, and graphs such as forest plots.

## Results

### Searching results

In this systematic review and meta-analysis, 1,075 articles were found from databases, organizational websites, and references of references in the search strategy. Of the total search results, 712 were excluded due to duplication, and 230 articles were excluded due to differences in study setting and study design (19–26). In addition to the above, 87 articles were screened out due to the difference in population (27–32), 20 articles were screened out because the study participants were the general population (33–36), and 15 articles were screened out because the searched articles were conducted at different studies design (34, 37, 38). Finally, exactly 11 cohort studies were included and followed in this study.

### Characteristics of the included studies

Eleven studies involving 4,563 schoolchildren were followed until the end of the study period to measure the outcome. The included studies were conducted in four regions and one city administration of Ethiopia. These were six studies (*n* = 6) (39–45) in the Amhara region, one (*n* = 1) (46) in the Oromia region, one (*n* = 1) (33) in the Tigray region, one (*n* = 1) (47) in theBenishangul-Gumuz region, and one (*n* = 1) (48) in Addis Ababa City Administration. The studies with the smallest and largest sample sizes were 271 and 721 from the Benishangul-Gumuz and Amhara regions, respectively. The schoolchildren were followed up for 3 to 14.25 years with 920–1,144.57 child-years of OI-free observations in the primary studies (Table 1).

**Table 1:** The characteristics of the included studies among Ethiopian children on ART.

Sn	Authors	Publication year	Region	Cases	Sample size	PYO	Incidence	Follow-up period
1	Endalk Birrie Wondifraw et al. (39)	2022	Amhara	130	341	1,802.4	6	12.5
2	Mamaru Wubale Melkamu et al. (40)	2020	Amhara	129	408	1,144.57	9.7	14.25
3	Dagnaw Amare Mequanente et al. (41)	2022	Amhara	219	389	5,214.29	4.2	5
4	Ermias Sisay Chanie et al. (42)	2021	Amhara	87	349	1,573.24	5.53	10
5	Sualiha Gebeyaw Ayalaw et al. (43)	2015	Amhara	52	271	1,100.5	4.9	4
6	Yihun Mulugeta Alemu et al. (44)	2016	Amhara	79	421	1,854	4.2	5
7	Aklilu Endalamaw et al. (45)	2018	Amhara	34	352	1,294.7	2.63	12
8	Masino Tessu Beshir et al. (46)	2022	Oromia	63	428	3,328.66	6.03	4
9	Zekarias Gessesse Arefaine et al. (33)	2022	Tigray	69	317	920	7.5	3
10	Ayinalem Alemu et al. (48)	2012	Addis Ababa	146	566	2,140.08	6.82	5
11	Fassikaw Kebede et al. (47)	2022	Benishangul-Gumuz	63	721	1,075.1	5.86	10

### The pooled onset of opportunistic infections

**Figure 2:**
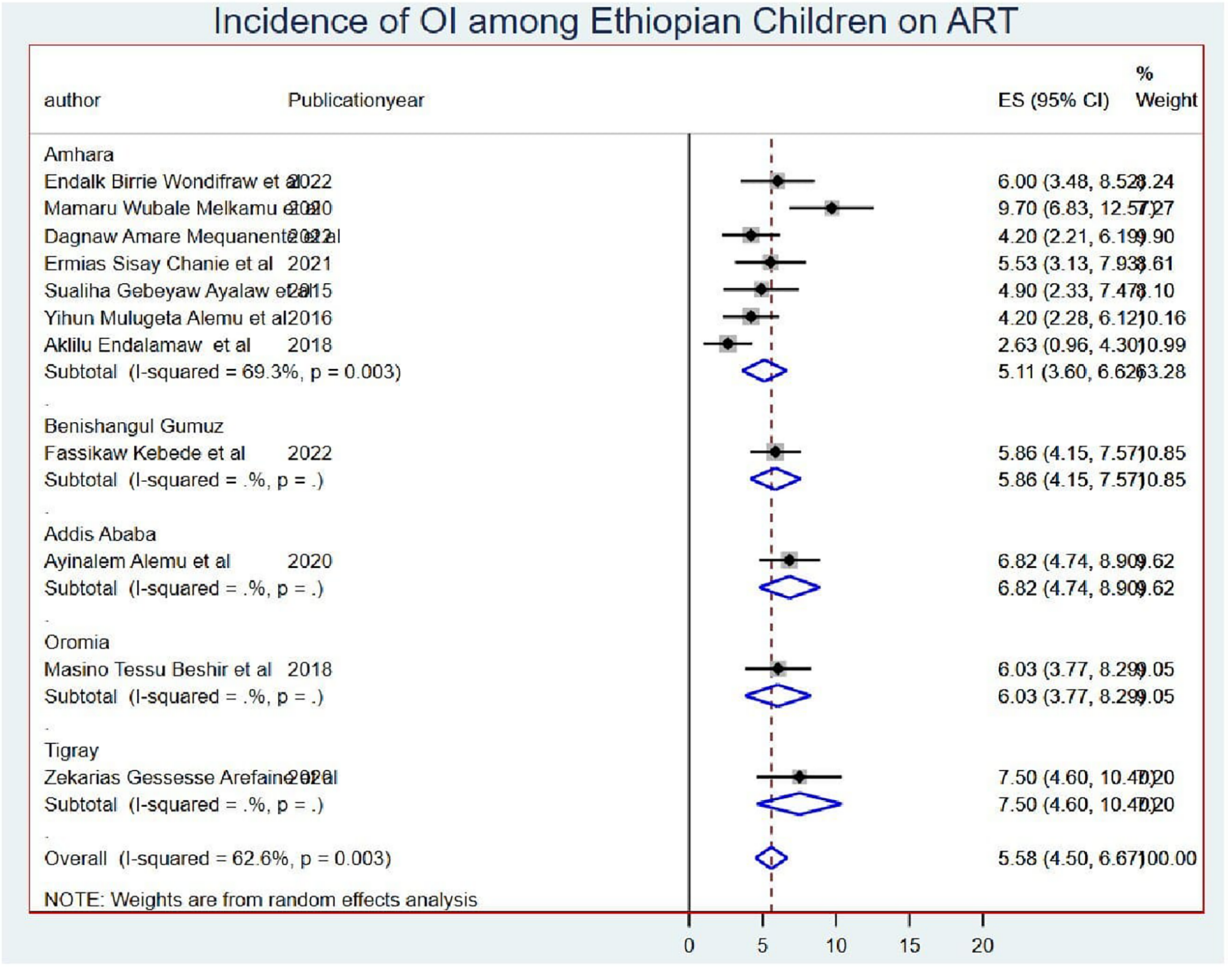
Incidence of OIs among Ethiopian children living with HIV and who are on ART.

The overall pooled onset of OIs among schoolchildren on ART was 5.58 (95% CI: 4.50, 6.67) per 100 child-years of OI-free observations in Ethiopia (Figure 2).

### Subgroup analysis

**Figure 3:**
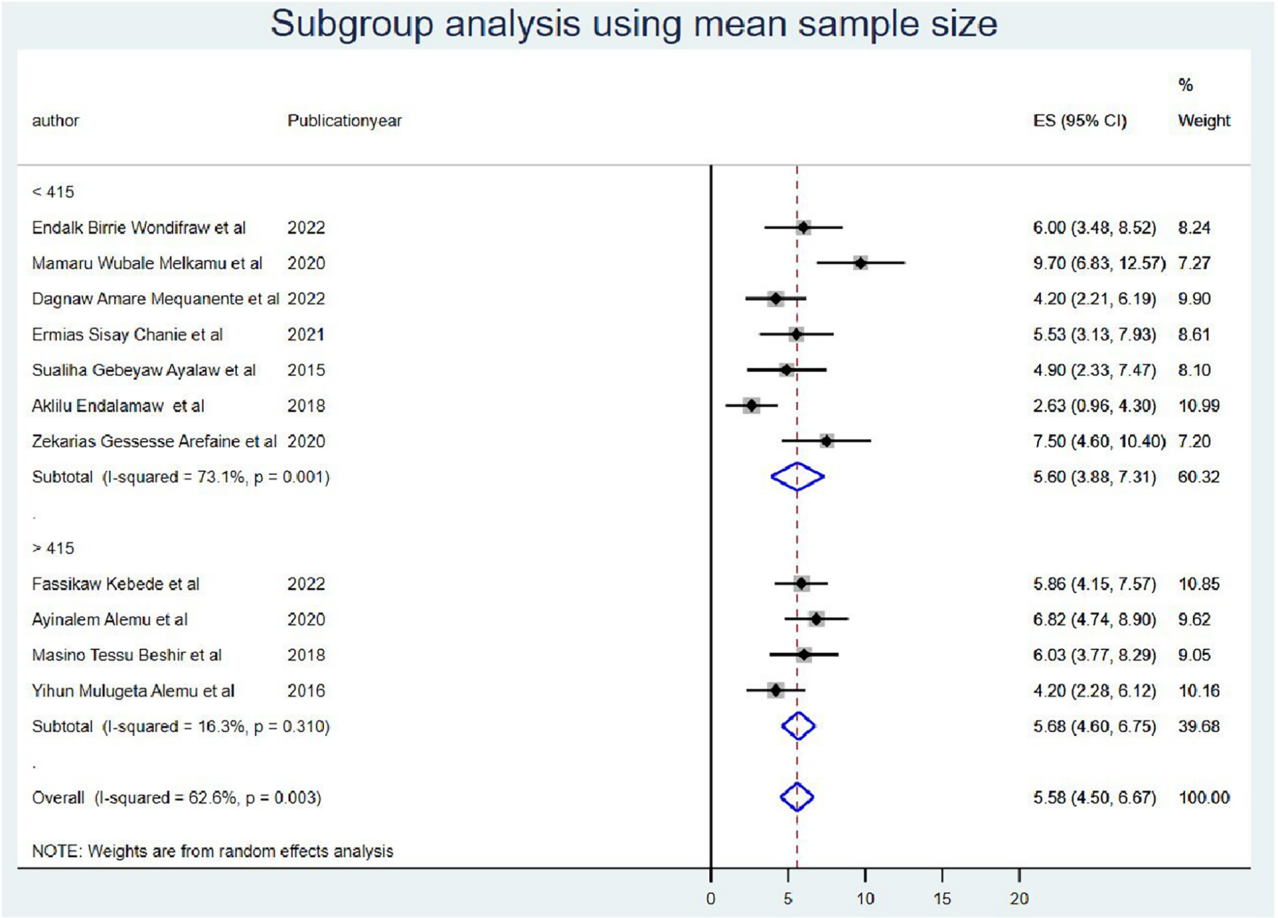
Subgroup analysis using mean sample size for OIs among Ethiopian children on ART.

**Figure 4:**
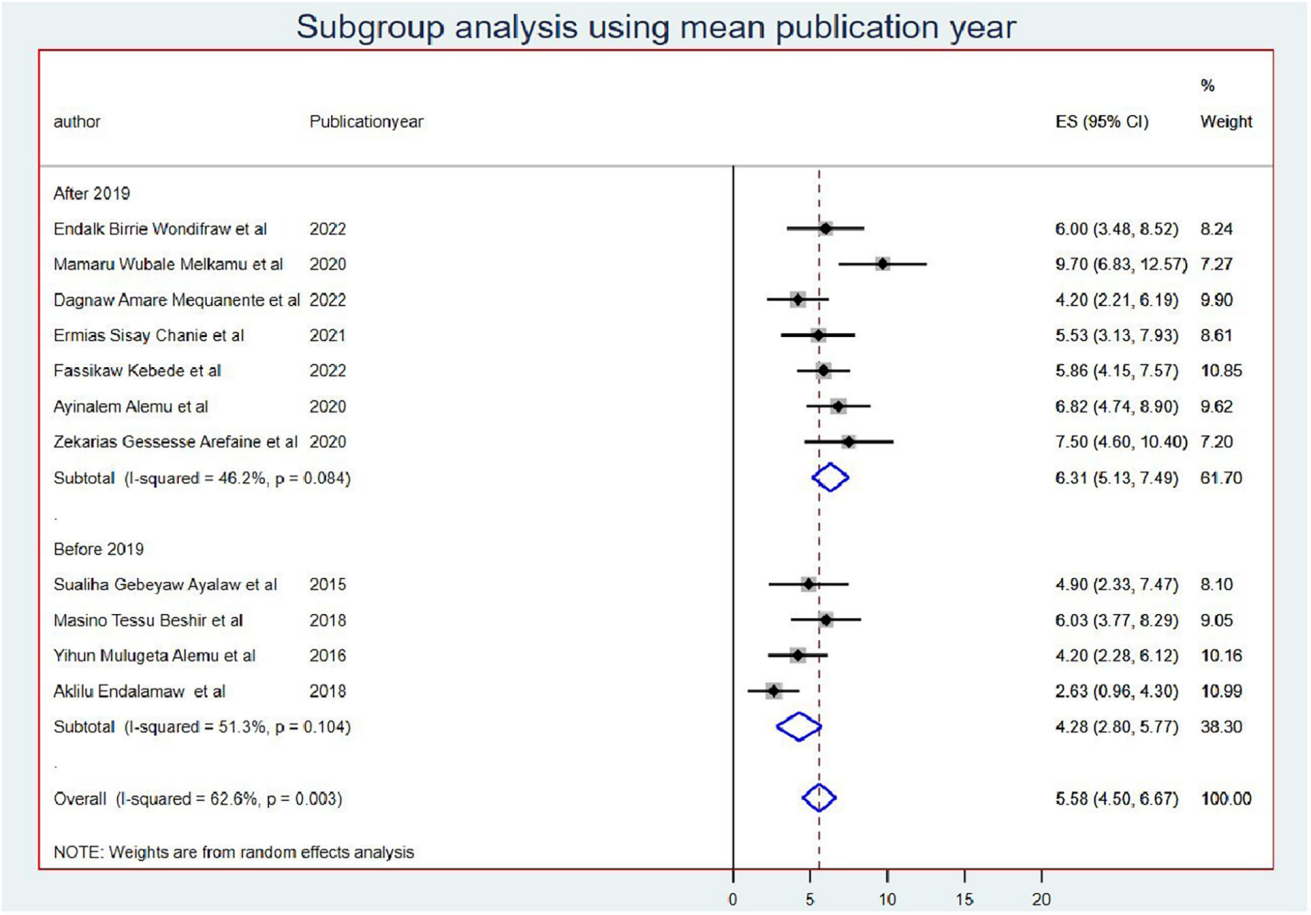
Subgroup analysis using mean publication year of OIs among Ethiopian children on ART.

The subgroup meta-analysis was computed using mean sample size (sample size for those whose sample size was less than the mean (<415) *I*^2^ = 73.1 and (>415) *I*^2^ = 16.3 with a *p*-value of <0.0001) (Figure 3) and publication year (those published before 2019 *I*^2^ = 51.3 with a *p*-value of <0.001 and after 2019 *I*^2^ = 46.2 with a *p*-value of <0.08) (Figure 4).

**Figure 5:**
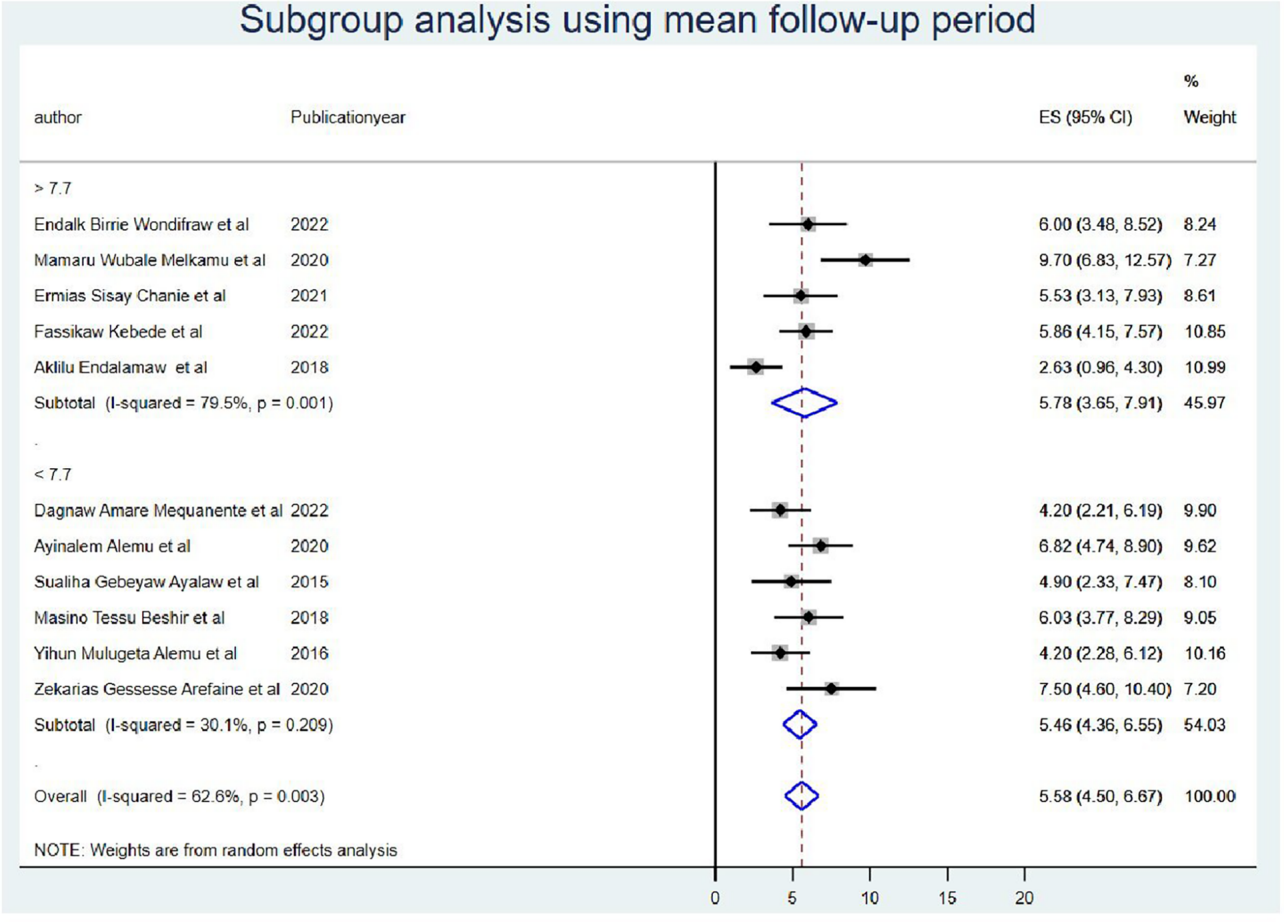
Subgroup analysis using mean follow-up period of OIs among Ethiopian children on ART.

Furthermore, the subgroup analysis was performed by taking the duration of the follow-up period as less than the mean follow-up years (<7.7 years) *I*^2^ = 30.1 with a *p*-value of 0.21 and greater than the mean follow-up years (>7.7 years) *I*^2^ = 79.5 with a *p*-value of <0.000) (Figure 5). Finally, the source of heterogeneity was not explained by subgroup meta-analysis.

### Meta-regression

In random-effects meta-regression, the year of publication and sample size were used as covariates. According to the findings, sample size (*p* = 0.86) and publication year (*p* = 0.80) did not affect heterogeneity (Table 2).

**Table 2:** The meta-regression of the included studies among Ethiopian children on ART.

logrr	Coefficient	Std. Err.	*t*	*p* > (t)	95% CI
Sampsize	0.000483	0.0027405	0.18	0.864	−0.0058365, 0.0068025
Publication year	0.0414859	0.1591993	0.26	0.801	−0.3256284, 0.4086002
Cons	−82.37613	320.9607	−0.26	0.804	657.7605

logrr, log relative risk; Std. Err., standard error.

### Publication bias

**Figure 6:**
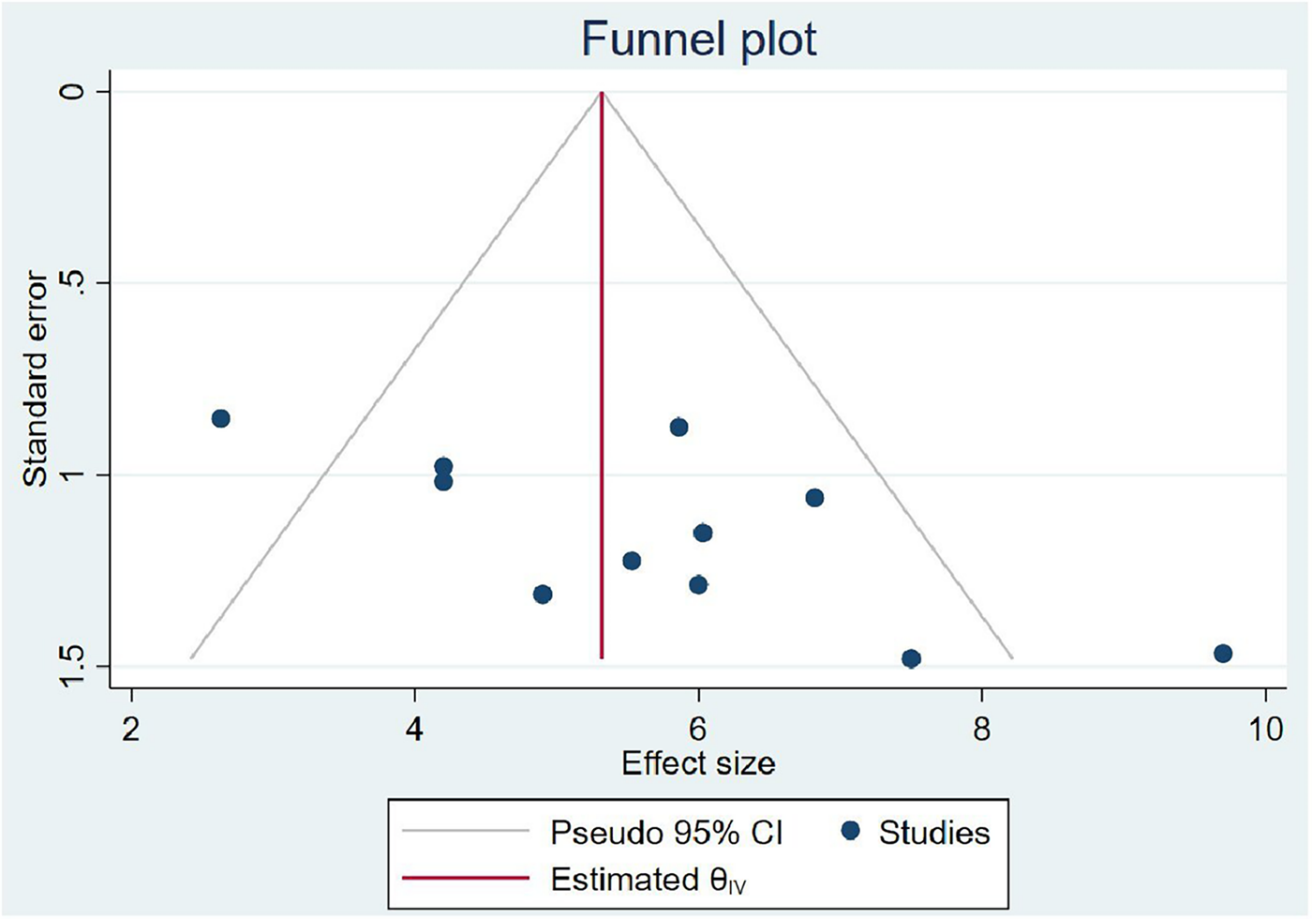
Funnel plot of OIs among Ethiopian children on ART.

In computing the publication bias, the funnel plot revealed that the small study had effects on the presence of heterogeneity of OIs among Ethiopian schoolchildren on ART (Figure 6) because the scatter plots were asymmetrical and located at the top. To overcome the subjective view of the funnel plot, the Egger linear regression test was computed and confirmed as there is no publication bias introduced (*p* = 0.00) (Table 3). Finally, a trim and fill analysis was performed to break the tie between the funnel plot and the Egger test, and it demonstrated that publication was not the source of heterogeneity for OIs among schoolchildren on ART in Ethiopia.

**Table 3:** Trim and fill analysis of OIs among Ethiopian children on ART.

Studies	Effect size	95% CI
Observed	5.581	4.504, 6.657
Observed + imputed	5.581	4.504, 6.657

### Predictors of OIs among school children on ART

To identify the predictors of OIs among schoolchildren on ART, baseline WHO stage, adherence, residence, follow-up duration, viral load, history of using prophylaxis, and parental status were considered. Finally, children's residence, ART adherence, and parents’ status were identified as significant predictors of OIs among schoolchildren on ART in Ethiopia.

**Figure 7:**
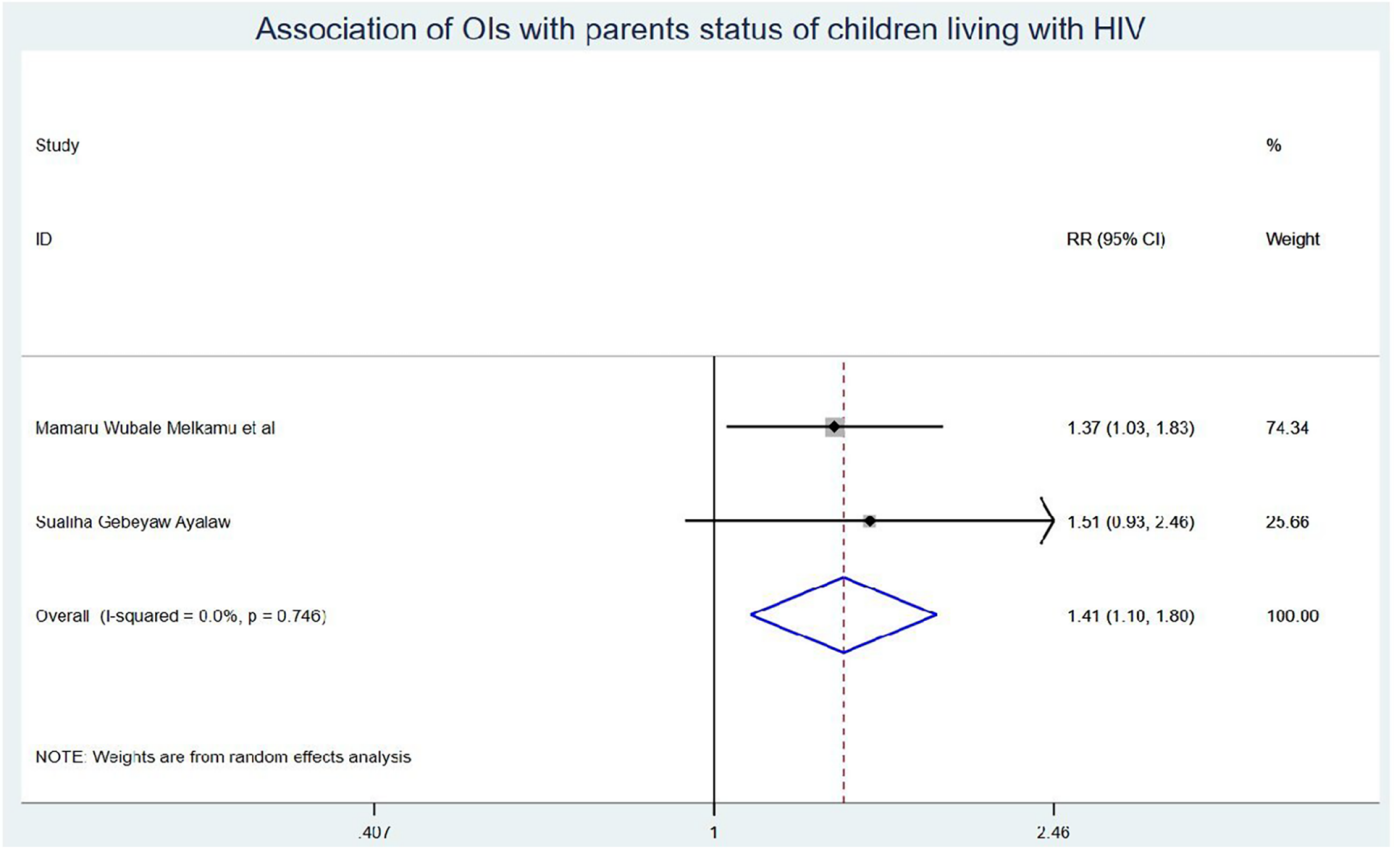
Association of OIs with parents status of Ethiopian children living with HIV.

**Figure 8:**
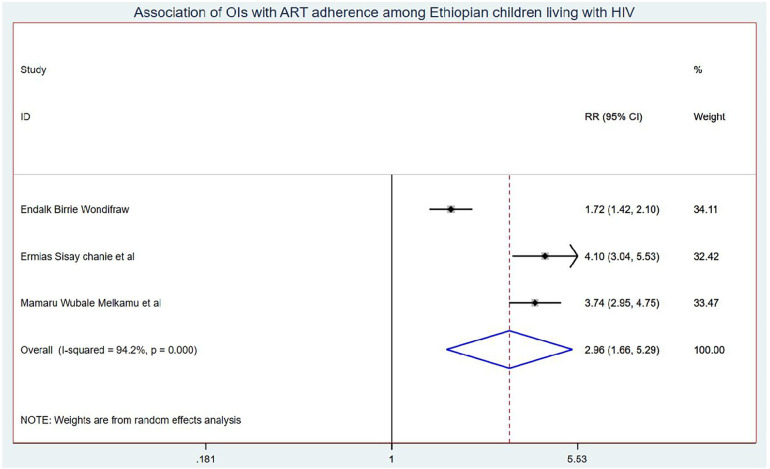
Association of OIs with ART adherence Ethiopian children living with HIV.

**Figure 9:**
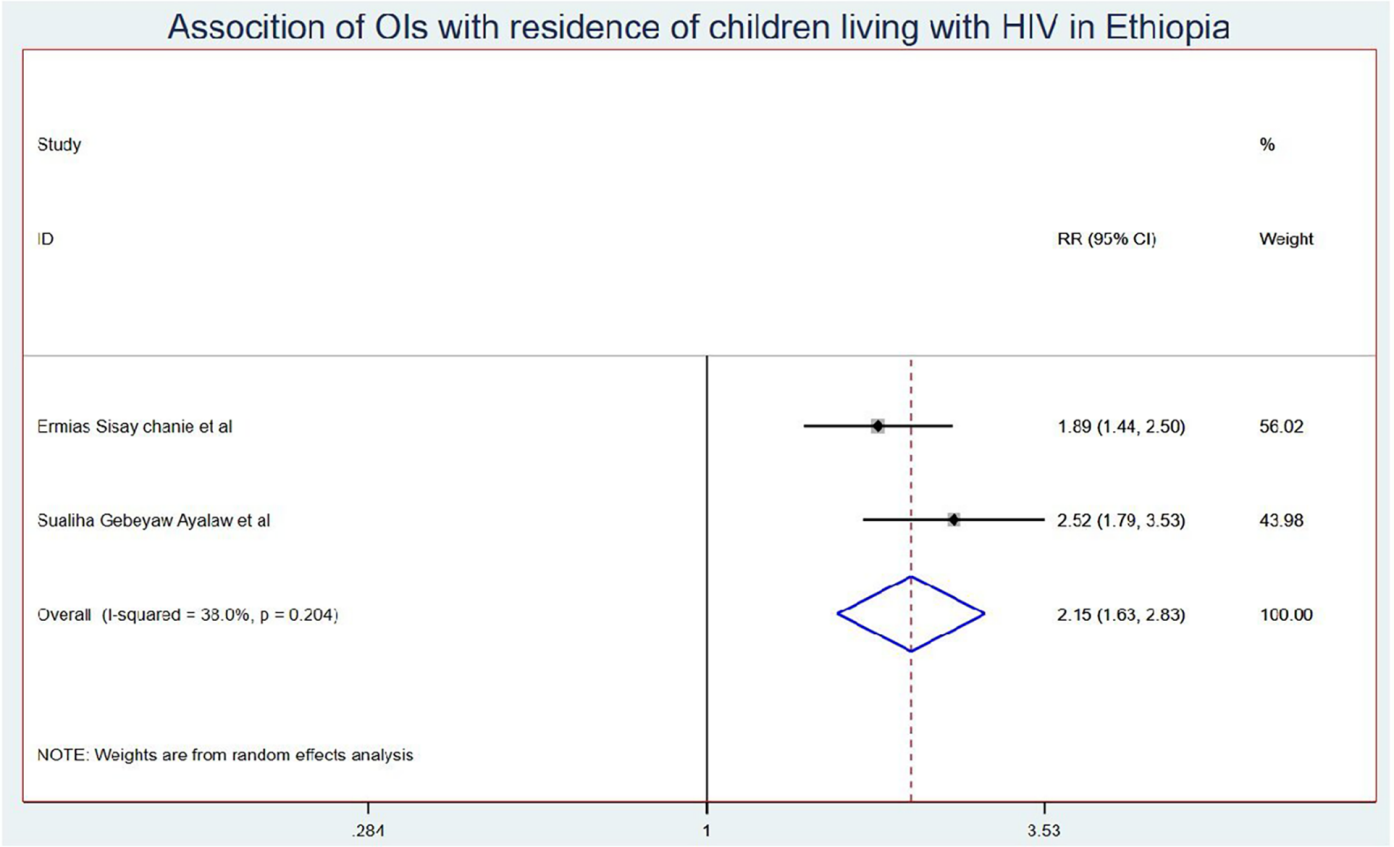
Association of OIs with residence of Ethiopian children living with HIV.

Those schoolchildren whose one or both parents died were1.41 (95% CI: 1.10, 1.80) times higher chance of getting OIs when compared with those children who had parents (Figure 7). Turning to ART adherence, children who had poor ART adherence had a 2.96 (95% CI: 1.66, 5.29) times higher chance of experiencing OIs than children who had good ART adherence (Figure 8). As a last point, the chance of experiencing OIs among schoolchildren who were living in rural was 2.15 (95% CI: 1.63, 2.83) times higher than their corresponding counterparts (Figure 9).

## Discussions

The included primary studies were 11 having 4,563 schoolchildren and were published between 2015 and 2022. The studies were conducted in a cohort study design having the smallest and largest sample sizes of 271 to 721, respectively.

The overall onset of OIs among schoolchildren on ART was 5.58 infections (95% CI: 4.50, 6.67) per 100 child-years of OI-free observation in Ethiopia. This result is too high to end the HIV/AIDS epidemic as a public health threat by 2030 because OIs are infections that occur more frequently and are more severe in people with weakened immune systems which is again a sign of ART failure (49). This might be because most Ethiopians live in rural in which healthcare facilities are limited access, and individuals in rural areas may face challenges in adhering to antiretroviral therapy (ART) due to factors such as limited availability of medication, transportation barriers, and stigma associated with HIV leading to poor adherence to ART. These can weaken the immune system, making individuals more susceptible to opportunistic infections. This finding is greater than the study finding of Thailand (25.6 per 1,000 person-years) (50). This might be due to the presence of advanced community awareness in Thailand made them have greater ART adherence than Ethiopians and led to higher OIs in Ethiopia than in Thailand (51). On the other hand, the onset of OIs in Ethiopia is lower than the study findings conducted in lower- and middle-income countries, which is greater than 5% (52). This might be due to the difference in the time of ART initiation secondary to the community awareness and treatment approach. This means that the included studies of this systematic review and meta-analysis were conducted after the test and treat principle of ART initiation was launched, and this in turn led the patients to have better outcomes sooner than the patients started after they developed advanced HIV stages.

The onset of OIs was lower than the Asia study findings of 28.8 infections per 100 child-years (53). This could be due to a difference in the time used to measure the onset of OIs in children on ART, as our study findings incorporate the most recent studies in which study participants became more aware of the importance of having regular ART follow-up with good ART adherence. However, this finding is still critical in combating the negative effects of HIV/AIDS on the quality and survival of children. Because of the emergence of opportunistic infections among children, living with HIV and on ART is the possible method to demonstrate the absence of the intended impact of ART on the virus for children, both directly and indirectly, because of one or more cause(s). As a result, all stakeholders should participate in ensuring that children have regular follow-ups with good ART adherence, particularly for those who are from rural areas.

Those study participants whose either or both parents died had a 1.41 (95% CI: 1.10, 1.80) times higher chance of getting OIs when compared with those children having one or both of their parents. This could be because orphan children were not able to start ART as early as recommended; they may have experienced significant immunosuppression and opportunistic infections before getting on treatment, and without the consistent support and monitoring of parents, they may struggle with adherence to their ART regimen. Missed doses can allow viral replication and opportunistic infections to occur (54). In addition, children are more likely to accept information such as taking daily ART doses to have good ART adherence and receiving regular follow-ups from parents than other guardians/caregivers (55). Hence, caregivers should approach children in a friendly manner to have regular follow-ups with good ART adherence, and healthcare workers should pay special attention to providing health education to those caregivers.

Children who have poor ART adherence had a 2.96 (95% CI: 1.66, 5.29) times higher chance of experiencing OIs than children having good ART adherence. This could be because good ART adherence is a key factor in controlling viral replication and improving patient immunity, whereas poor antiretroviral drug adherence in children is associated with a loss of sustained viral suppression, an increased risk of drug resistance, and disease progression that leads to opportunistic infection (56, 57).

The chance of experiencing OIs among Ethiopian children who were living in rural areas was 2.15 (95% CI: 1.63, 2.83) times higher than their corresponding counterparts. This could be because children from rural areas travel long distances to get access to ART. This may lead them to scarcity of transportation costs due to the lack of nearby treatment centers or distance from treatment centers, low level of awareness about the benefits and risks of adhering to ART care and treatment, and the social stigma associated with HIV/AIDS. In addition to the above, patients from rural areas often travel greater distances to receive care due to fewer healthcare facilities and specialized providers. They may face ART drug stock-outs that can lead to interruptions in ART access and adherence, lack of education, and poverty, which can lead to developed opportunistic infections (58).

## Conclusion and recommendations

Among Ethiopian schoolchildren living with HIV and on ART, three in every thirty-three were at risk of developing OIs. The combination of delayed ART access, adherence challenges, advanced disease, and other socioeconomic factors can all contribute to the increased risk of opportunistic infections among orphan children on ART. Targeted support and close clinical monitoring are important to overcome these challenges.

It is better to strengthen the access to healthcare to improve access to healthcare services in rural areas, including HIV testing, treatment, and monitoring. Ensure that schoolchildren living with HIV have regular access to healthcare providers who can monitor their condition, provide the necessary support, and strengthen community engagement to engage community members, schools, and local organizations in raising awareness about HIV, reducing stigma, and supporting schoolchildren living with HIV. Community involvement can help create a supportive environment for children affected by HIV.

There should be enhanced adherence support to implement interventions to improve ART adherence among schoolchildren living with HIV. This could include educational programs, reminder systems, peer support groups, and counseling services to help children and caregivers adhere to their medication regimens, and it is better to provide social support to establish social support programs for schoolchildren living with HIV who do not have parents or have limited family support. These programs could include mentorship, counseling, and assistance with basic needs such as food, shelter, and education.

Finally, there should be monitor and evaluation to track the progress of interventions aimed at reducing OIs among schoolchildren living with HIV since regular assessments can help identify gaps in services and guide future efforts to improve health outcomes.

## Data Availability

The original contributions presented in the study are included in the article/Supplementary Material, further inquiries can be directed to the corresponding author.
